# Proteinuria Is Independently Associated with Impaired Gallbladder Emptying in Biopsy-Proven Glomerular Disease with Preserved Kidney Function: A Case–Control Ultrasonographic Study

**DOI:** 10.3390/jcm15166267

**Published:** 2026-08-13

**Authors:** Simal Koksal Cevher, Hasan Tankut Koseoglu, Sabri Onur Ozden, Emre Cankaya, Ezgi Coskun Yenigun, Fatih Dede

**Affiliations:** 1 Department of Nephrology, Ankara Bilkent City Hospital, Ankara 06800, Türkiye; emrecnky@hotmail.com (E.C.); drezgi76@gmail.com (E.C.Y.); fatded@yahoo.com (F.D.); 2 Department of Gastroenterology, Ankara Bilkent City Hospital, Ankara 06800, Türkiye; tankutkoseoglu@gmail.com; 3 Family Medicine Clinic, Gulagac District State Hospital, Aksaray 68900, Türkiye; sabrionurozden@hotmail.com

**Keywords:** proteinuria, glomerulonephritis, gallbladder ejection fraction, gallbladder dysmotility, cholecystokinin, ultrasonography, nephrotic syndrome

## Abstract

**Background/Objectives**: Gallbladder emptying is primarily regulated by postprandial cholecystokinin release. In proteinuric glomerular diseases, renal loss of peptide hormones, hormone-binding proteins, or related regulatory proteins may affect biliary motility. However, gallbladder function has not been adequately investigated in individuals with proteinuric glomerular disease. This study evaluated the association between proteinuria and gallbladder emptying in patients with preserved kidney function and biopsy-proven glomerular disease. **Methods**: This single-center ultrasonographic case–control study with prospective participant enrollment and data collection included 106 participants: 52 patients with biopsy-proven proteinuric glomerular disease and 54 healthy controls without proteinuria. The proteinuric patient group consisted of individuals diagnosed with primary glomerulonephritis or amyloid A (AA) amyloidosis. Acute kidney injury, estimated glomerular filtration rate <60 mL/min/1.73 m^2^, cholelithiasis, liver disease, diabetes mellitus, previous upper gastrointestinal surgery, pregnancy, oral contraceptive use, and recent rapid weight loss were considered exclusion criteria. After an overnight fast of at least 8 h, fasting gallbladder volume was measured by ultrasonography and recorded as baseline volume (V0). Gallbladder volume was measured again 45 min after stimulation with a standardized 40 g chocolate meal and recorded as postprandial volume (V45). Gallbladder volume was calculated using the ellipsoid formula, and gallbladder ejection fraction (GBEF) was calculated as [(V0 − V45)/V0] × 100. GBEF <40% was used as a predefined clinical threshold for impaired gallbladder emptying. In the primary analysis, the association between proteinuria status and GBEF as a continuous outcome was evaluated using a multivariable linear regression model adjusted for age, sex, body mass index, and family history of gallstones. Impaired gallbladder emptying, operationally defined as GBEF <40% for the supportive secondary binary outcome, was examined using multivariable logistic regression model adjusted for the same covariates. **Results**: Compared with controls, proteinuric patients had significantly higher postprandial gallbladder volume at 45 min: 15,614.04 ± 9148.35 mm^3^ versus 10,346.89 ± 5254.17 mm^3^ (*p* = 0.0003). GBEF was significantly lower in the proteinuria group than in healthy controls: 41.76% ± 19.64 versus 53.10% ± 20.22; mean difference, −11.34 percentage points (95% confidence interval, −19.02 to −3.66; *p* = 0.0042). Impaired gallbladder emptying was more frequently observed in the proteinuria group: 48.1% versus 27.8% (*p* = 0.031). In unadjusted analysis, the presence of proteinuria was associated with a 2.41-fold increase in the odds of impaired gallbladder emptying. This association remained statistically significant after adjustment for age, sex, and body mass index: adjusted odds ratio, 2.95 (95% confidence interval, 1.17–7.47; *p* = 0.022). Among proteinuric patients, GBEF did not differ significantly according to nephrotic versus non-nephrotic proteinuria, serum albumin level, serum total protein level, or histopathological diagnostic subgroup. **Conclusions**: In individuals with preserved kidney function and biopsy-proven glomerular disease, proteinuria was associated with impaired postprandial gallbladder emptying, and this association persisted after multivariable adjustment. These findings suggest that gallbladder dysmotility may represent a potentially relevant functional feature of proteinuric kidney disease; however, its biological basis and clinical consequences remain to be established. Prospective studies incorporating cholecystokinin measurements, duration of proteinuria, and longitudinal clinical follow-up are needed to clarify these issues.

## 1. Introduction

The gallbladder is a dynamic component of enterohepatic physiology that enables the regulated delivery of concentrated bile into the duodenum during the postprandial period. Cholecystokinin (CCK) is the principal hormonal regulator of gallbladder contraction triggered by the arrival of fat- and protein-rich nutrients in the duodenum. CCK is a peptide gastrointestinal hormone secreted primarily by enteroendocrine I cells in the duodenum and jejunum; it initiates gallbladder contraction by stimulating CCK1 receptors on gallbladder smooth muscle cells, while simultaneously contributing to a reduction in sphincter of Oddi tone and thereby facilitating the passage of bile into the intestinal lumen. The contractile effect of CCK on the gallbladder is not limited to a direct receptor-mediated response at the smooth muscle level, but is also supported through interactions with intrinsic and extrinsic cholinergic neural pathways [1,2]. Therefore, the CCK–cholinergic axis plays a central role in maintaining physiological gallbladder emptying [2].

Impaired gallbladder motility is clinically important because of its association with bile stasis and biliary complications. Gallstone disease is associated with highly morbid conditions such as cholecystitis, cholangitis, obstructive jaundice, and pancreatitis; its pathogenesis involves the combined contribution of lithogenic bile composition, crystal nucleation, and gallbladder dysfunction. In addition to the balance among cholesterol, bile acids, and phospholipids in bile, impairment of gallbladder emptying, absorptive, and secretory functions also predisposes to stone formation [3]. Genetic and intestinal factors also contribute to the pathogenesis of gallstone disease [4,5,6].

Gallbladder dysmotility has been investigated in various conditions, including obesity, pregnancy, diabetes mellitus, parenteral nutrition, reduced oral intake, medication use, and autonomic nervous system dysfunction. Ultrasonography (USG) and cholescintigraphy are used for the assessment of gallbladder motility, and a fatty meal is most commonly preferred as the physiological stimulus. Although ultrasonography is operator-dependent, it represents a practical option for measuring gallbladder volume and calculating gallbladder ejection fraction because it is low-cost, easy to perform, radiation-free, and readily repeatable [7].

Proteinuric glomerular diseases are characterized by pathological urinary protein loss resulting from impaired selective permeability of the glomerular filtration barrier. Nephrotic syndrome is classically defined by nephrotic-range proteinuria (>3.5 g/day), hypoalbuminemia, edema, and frequently hyperlipidemia. In adults, the major primary glomerular etiologies include membranous nephropathy, focal segmental glomerulosclerosis (FSGS), and minimal change disease (MCD), whereas immunoglobulin A (IgA) nephropathy may less commonly present with nephrotic syndrome or with a mixed nephritic–nephrotic phenotype [8]. During proteinuric states, not only albumin but also low- or intermediate-molecular-weight proteins, peptide-based molecules, and various binding proteins may be lost in the urine [8]. Therefore, it is biologically plausible that proteinuric states may affect gallbladder emptying through the CCK axis or related neurohormonal regulatory mechanisms, although this remains a hypothesis requiring direct confirmation.

Although data on the relationship between kidney disease and the biliary system remain limited, it has been reported that the uremic milieu, autonomic dysfunction, nutritional changes, and hormonal imbalances in chronic kidney disease may affect gallbladder motility [3,9]. Data specifically focusing on proteinuria are even more limited; epidemiological findings indicating an increased risk of gallstone development in severe proteinuria suggest that proteinuria may be an independent risk indicator for gallstone disease [9]. Gallbladder dysmotility may represent one of the intermediate steps in this potential association.

Gallbladder ejection fraction (GBEF) is a practical functional parameter used to assess gallbladder contractility. In ultrasonographic studies performed with fatty meal stimulation, GBEF values below 35–40% at 45–60 min are generally interpreted as indicative of reduced contractility, and a 40% cut-off value may be used to distinguish patients from controls [10]. In the current literature, data directly evaluating gallbladder motility in individuals with proteinuric glomerular disease, preserved kidney function, and exclusion of major confounding factors such as cholelithiasis and diabetes mellitus are extremely limited. Therefore, this study aimed to compare gallbladder motility between healthy controls and patients with preserved kidney function who had proteinuria due to biopsy-proven primary glomerulonephritis or AA amyloidosis, and to evaluate the relationship of proteinuria status and severity with GBEF.

## 2. Materials and Methods

### 2.1. Study Design, Setting, and Participants

This was a single-center, controlled, observational case–control study conducted within the same nephrology clinical research program in Ankara, Türkiye, to evaluate gallbladder motility in individuals with biopsy-confirmed proteinuric glomerular disease and healthy controls. Participant enrollment and study-specific data collection were carried out through the Department of Nephrology. Participants were prospectively enrolled, and study-specific clinical, laboratory, and ultrasonographic data were prospectively collected during two distinct enrollment periods: 1 March 2016 to 31 May 2017 and 22 January 2026 to 4 May 2026. The first enrollment period included 33 proteinuric patients and 23 healthy controls, whereas the second enrollment period included an additional 19 proteinuric patients and 31 healthy controls. These two enrollment periods are reported solely to clarify the chronology of participant recruitment and do not represent separate study cohorts, study phases, or statistical strata; all participants were analyzed within the same case–control framework according to their proteinuria status. The final study population therefore comprised 106 participants, consisting of 52 proteinuric patients followed in the nephrology outpatient clinic and diagnosed by kidney biopsy with primary glomerulonephritis or AA amyloidosis, and 54 healthy volunteers who had no proteinuria or known kidney disease and served as the control group.

Written informed consent was obtained from all participants in both enrollment periods before their inclusion in the study. The first enrollment period was completed before the institutional ethics committee framework was extended, on 8 November 2019, to include non-interventional clinical studies of this type and therefore did not require retrospective ethics committee approval. For the second enrollment period, prospective ethics committee approval was obtained before any participant was enrolled. The ethics approval framework and informed consent procedures applicable to both enrollment periods are described in detail in the Institutional Review Board Statement and Informed Consent Statement below.

The healthy controls were recruited as an unmatched physiological reference group rather than as individually matched comparators. The purpose of this group was to characterize gallbladder emptying under the same standardized fasting, meal-stimulation, ultrasonographic, and measurement conditions in individuals with neither proteinuria nor known kidney disease. Individual matching was not incorporated into the original study design because the availability and covariate distribution of eligible patients with biopsy-proven proteinuric glomerular disease could not be predicted reliably during recruitment. Consequently, all eligible participants who fulfilled the predefined criteria were retained. This approach maximized the use of the available data but allowed baseline imbalances between the patient and control groups.

The proteinuric patient group consisted of 45 individuals with primary glomerulonephritis (idiopathic membranous nephropathy, minimal change disease, focal segmental glomerulosclerosis, or IgA nephropathy) and 7 patients with amyloid A (AA) amyloidosis.

*Exclusion Criteria*: To reduce potential confounding factors that could affect gallbladder motility, individuals with acute kidney injury, estimated glomerular filtration rate (eGFR) <60 mL/min/1.73 m^2^, cholelithiasis, previous upper gastrointestinal surgery, known liver disease, diabetes mellitus, pregnancy, oral contraceptive use, or a history of recent rapid weight loss were excluded from the study. These exclusion criteria were applied to minimize potential confounding by factors known or suspected to influence gallbladder volume and gallbladder ejection fraction, including uremia or advanced kidney dysfunction, diabetic autonomic neuropathy, pre-existing gallstone disease, liver disease, pregnancy- or oral-contraceptive-related hormonal effects, and recent rapid weight loss.

Hypertension (HT) was not considered an exclusion criterion. Because the patient population was defined by biopsy-proven proteinuric glomerular disease, hypertension could represent a pre-existing comorbidity, a consequence of renal disease, or a marker of disease severity. Excluding all participants with hypertension would have selectively removed a substantial and clinically representative proportion of the target population.

Proteinuria status was defined according to the spot urine protein-to-creatinine ratio (UPCR). Absent proteinuria was defined as UPCR <0.2 g/g creatinine. Among proteinuric patients, non-nephrotic proteinuria was defined as UPCR ≥0.2 and ≤3.5 g/g creatinine, whereas nephrotic-range proteinuria was defined as UPCR >3.5 g/g creatinine. All proteinuric patients included in the study were evaluated within the first 3 months after diagnosis. Therefore, the proteinuric cohort was considered a relatively homogeneous patient group composed of newly diagnosed or early-stage cases. This approach was intended to minimize the potential impact of disease-duration-related heterogeneity on comparisons between proteinuric patients and controls, as well as on subgroup analyses performed within the proteinuric patient group according to proteinuria severity.

All participants were evaluated for demographic characteristics, clinical variables, and laboratory parameters potentially associated with gallbladder function. Patients and controls were compared with respect to fasting gallbladder volume (V0), 45 min postprandial gallbladder volume (V45), and gallbladder ejection fraction (GBEF). In addition, within the proteinuric patient group, gallbladder volume and emptying parameters were compared according to proteinuria severity, defined as non-nephrotic versus nephrotic-range proteinuria, and according to renal biopsy diagnosis subgroups.

### 2.2. Clinical and Laboratory Assessment

Venous blood samples were obtained from all participants after a fasting period of at least 8 h. Laboratory evaluation included fasting glucose, serum creatinine, total protein, albumin, total cholesterol, triglycerides, high-density lipoprotein cholesterol, low-density lipoprotein cholesterol, aspartate aminotransferase, alanine aminotransferase, γ-glutamyltransferase, total bilirubin, direct bilirubin, C-reactive protein (CRP), complete urinalysis, and spot urine protein-to-creatinine ratio. Kidney function was assessed using the estimated glomerular filtration rate (eGFR), calculated from serum creatinine according to the Chronic Kidney Disease Epidemiology Collaboration (CKD-EPI) equation.

### 2.3. Gallbladder Ultrasonography and Motility Assessment

Following clinical evaluation and the collection of blood and urine samples for laboratory analyses, gallbladder ultrasonography (USG) was performed in all participants on the same study day. All USG examinations were carried out by the same gastroenterology specialist using the same ultrasonography device. The examiner was blinded to participant group allocation during image acquisition and gallbladder measurements. Measurements were performed with a General Electric LOGIQ A5 Pro device equipped with a 3.5 MHz convex transducer. For each participant, fasting and postprandial gallbladder measurements were obtained during a single examination session. No separate-day repeat examination was performed. Because no second examiner participated in image acquisition or measurement and duplicate blinded remeasurements were not performed, neither interobserver nor formal intraobserver reproducibility was assessed.

Gallbladder volume was assessed at baseline after a fasting period of at least 8 h and again after a standardized meal stimulus. A 40 g chocolate meal was used as the standardized stimulus. This meal contained 194 kcal, with a nutritional composition of 58.5% carbohydrate, 26.2% fat, and 4% protein. Chocolate was selected as a practical, readily available, palatable, mixed-nutrient stimulus expected to elicit endogenous cholecystokinin release and physiological postprandial gallbladder contraction. After consumption of the standardized chocolate meal, a second USG measurement was performed at 45 min, the time point at which maximal gallbladder contraction was expected. This protocol was intended to provide standardized physiological stimulation for comparisons between the study groups and was not regarded as a substitute for a formally validated sincalide-stimulated hepatobiliary scintigraphy protocol.

Maximum gallbladder width, length, and depth were measured in the fasting state and at 45 min postprandially. Gallbladder volume was calculated using the ellipsoid formula [11]:

V = (π/6) × width × length × depth.

Fasting gallbladder volume was defined as V0, whereas the 45 min postprandial gallbladder volume was defined as V45. Gallbladder ejection fraction (GBEF) was calculated from fasting and postprandial volumes according to the following formula [3]:

GBEF (%) = [(V0 − V45)/V0] × 100.

A GBEF value <40% was accepted as a clinically meaningful threshold for impaired gallbladder emptying [10].

*Outcomes of the study*: The primary outcome variable was GBEF expressed as a percentage. The supportive secondary outcome was impaired gallbladder emptying (GBEF <40%). Additional exploratory analyses evaluated V0, V45, and gallbladder motility according to proteinuria severity, serum protein parameters, and histopathological diagnosis.

### 2.4. Statistical Analysis

All statistical analyses were performed using R software, version 4.5.2 (R Core Team), within the RStudio 2026.07.0 integrated development environment provided by Posit. Continuous variables were expressed as mean ± standard deviation or median and interquartile range, depending on their distribution. Categorical variables were presented as counts and percentages. The normality of continuous variables was assessed using the Shapiro–Wilk test, histograms, and quantile–quantile (Q–Q) plots. Homogeneity of variances was evaluated using Levene’s test.

For two-group comparisons, the independent-samples Student’s *t*-test was used for approximately normally distributed continuous variables with homogeneous variances, whereas Welch’s *t*-test was used when the normality assumption was satisfied but the homogeneity of variance assumption was not met. For two-group comparisons of non-normally distributed continuous variables, the Mann–Whitney U test was used. For comparisons involving more than two groups, one-way analysis of variance or the Kruskal–Wallis test was preferred according to the distributional characteristics of the variables. Categorical variables were compared using Pearson’s chi-square test, whereas Fisher’s exact test was used when expected cell counts were low.

The association between proteinuria status and GBEF (%) was evaluated using a multivariable linear regression adjusted for age, sex, body mass index (BMI), and family history of gallstones (GBstone.FH). The supportive secondary binary outcome variable was impaired gallbladder emptying, defined as GBEF < 40%. Separate exploratory univariable logistic regression models were used to describe the unadjusted associations of selected demographic, clinical, and laboratory variables with GBEF < 40%. These analyses were performed for descriptive and hypothesis-generating purposes and were not used as a screening procedure for covariate selection. The relationship between the proteinuria status and impaired gallbladder emptying was examined using multivariable logistic regression analysis. Because 40 participants had GBEF <40%, the model was limited to 4 predictor parameters to maintain a conventional ratio of 10 events per parameter. In this parsimonious model, GBEF <40% was specified as the dependent binary outcome, proteinuria status as the primary explanatory variable, and age, sex, and BMI as adjustment covariates [logit{P(GBEF<40%)} = *β*_0_ + *β*_1_ *group* + *β*_2_ *age* + *β*_3_ *sex* + *β*_4_ *BMI*]. To assess the robustness of the findings to additional adjustment for a clinically relevant biliary risk factor, an expanded sensitivity model was also fitted by adding family history of gallstones to the parsimonious model. Age, sex, BMI, and family history of gallstones (GBstone.FH) were included as potential confounding variables in both the multivariable linear regression model and the multivariable logistic regression model. Covariates were selected according to clinical relevance, observed baseline differences between the study groups, and model parsimony rather than univariable statistical significance. No forward, backward, stepwise, or *p*-value-driven variable-selection procedure was used.

Because hypertension was substantially more prevalent in the proteinuria group and could function as either a confounder or a disease-related intermediate or severity marker, an extended sensitivity model was fitted by adding HT status to the primary multivariable linear regression model:

*GBEF* (%) = *β*_0_ + *β*_1_ *group* + *β*_2_ *age* + *β*_3_ *sex* + *β*_4_ *BMI* + *β*_5_ *GBstone.FH* + *β*_6_ *HT* + *ε*.

An exploratory receiver operating characteristic (ROC) curve analysis was performed to describe the cohort-specific discrimination of GBEF (%) between the proteinuria and control groups. This ROC analysis was planned as an exploratory discriminatory analysis distinct from the explanatory framework of the primary linear regression model; in this ROC analysis, GBEF (%) was not treated as an outcome variable but as a continuous marker whose discriminatory performance was evaluated. Whereas GBEF was treated as the outcome variable and proteinuria status as the main explanatory variable in the primary analysis, proteinuria status was used as the binary reference variable and GBEF (%) was used as the continuous marker in the ROC analysis. The area under the curve (AUC), sensitivity, and specificity were calculated. The Youden index was used to determine the cohort-specific optimal threshold for distinguishing proteinuric patients from healthy controls. In addition, a restricted ROC analysis was performed within the clinically relevant 35–45% GBEF threshold range for pathological gallbladder emptying. Accordingly, all operating points derived from the unrestricted and restricted ROC analyses should be interpreted solely as cohort-specific, data-driven exploratory values describing discrimination between proteinuric patients and healthy controls, and not as evidence of causality or as validated diagnostic, predictive, pathological, or clinically applicable cutoffs.

#### Sample Size and Power Considerations

No formal a priori sample size calculation was performed before participant enrollment. Because this was a single-center observational case–control study conducted in a clinically selected population of patients with biopsy-proven proteinuric glomerular disease and preserved kidney function, the final sample size was determined primarily by the availability of eligible participants who fulfilled the predefined inclusion and exclusion criteria during the study enrollment periods.

To evaluate the statistical sensitivity of the final sample for the prespecified primary continuous outcome, two complementary retrospective analyses were performed. Full formulas, assumptions, R code, and numerical calculations are provided in the Supplementary Material.

First, an unadjusted analysis was performed for the between-group comparison of mean GBEF. Mean GBEF was 53.10 ± 20.22% in the healthy control group and 41.76 ± 19.64% in the proteinuria group, corresponding to an unadjusted mean difference of 11.34 percentage points and a pooled standard deviation of 19.94 percentage points. The resulting standardized mean difference was Cohen’s d=0.569. With 54 healthy controls and 52 participants with proteinuria, a two-sided, equal-variance independent-samples *t*-test with an α level of 0.05 yielded an estimated post hoc power of 82.7% for detecting an unadjusted effect of the observed magnitude. Assuming equal allocation between groups and a true standardized effect equal to the observed Cohen’s d, the retrospectively estimated minimum sample size required to achieve at least 80% power was 50 evaluable participants per group. Both observed group sizes exceeded this threshold.

Second, because the primary inference was based on a multivariable linear regression model adjusted for age, sex, body mass index, and family history of gallstones, a regression-based analysis was performed for the adjusted proteinuria coefficient. The observed partial $R^{2}$ for proteinuria status was 0.07304, corresponding to a partial Cohen’s $f^{2}$ of 0.07879. Under the general linear model framework, with one tested regression coefficient, 100 residual degrees of freedom, and an α level of 0.05, this observed partial effect corresponded to an estimated post hoc power of 80.17%. For the fixed sample of 106 participants, the minimum detectable partial Cohen’s $f^{2}$ at 80% power was 0.07845, corresponding to a partial $R^{2}$ of 0.07275.

Assuming a true partial Cohen’s $f^{2}$ equal to the observed value, 1 tested regression coefficient, 5 explanatory variables, an α level of 0.05, a target power of 80%, and no missing or non-evaluable observations, the retrospectively estimated minimum total sample size was 106 participants. Direct integer-based verification showed that *N* = 105 would provide 79.77% power, whereas *N* = 106 would provide 80.17% power, confirming that 106 was the smallest integer total sample size meeting the target-power criterion under the stated assumptions.

These analyses were based on effect sizes observed in the final dataset and should not be interpreted as an a priori justification of the enrolled sample size or as evidence independent of the corresponding effect estimates and confidence intervals. Rather, they indicate that the final sample provided approximately 80% statistical sensitivity to detect unadjusted and adjusted associations of the magnitudes observed. The adjusted regression coefficient and its 95% confidence interval remain the primary statistical evidence.

The secondary binary outcome, impaired gallbladder emptying defined as GBEF <40%, was analyzed as a supportive outcome. Because the number of binary events was limited and dichotomization of GBEF reduces the information retained in the continuous outcome, this analysis was not used as the basis for sample size determination and was not considered definitive evidence on its own.

In all statistical analyses, a two-sided *p* value of <0.05 was considered statistically significant.

## 3. Results

### 3.1. Demographic and Clinical Characteristics of the Participants

A total of 106 participants were included in the study; 52 were in the proteinuria group and 54 were in the healthy control group. The mean age was 41.33 ± 8.86 years in the proteinuria group and 36.33 ± 11.22 years in the control group. The proteinuria group was significantly older than the control group [Welch’s *t*-test: *p* = 0.0124; 95% confidence interval (CI): 1.10–8.88] (Table 1). Therefore, age was considered a potential confounding variable in the multivariable regression models. In the proteinuria group, 24 patients (46.15%) were female, whereas 38 individuals (70.37%) in the control group were female. A statistically significant difference was observed between the groups in terms of sex distribution (Pearson’s chi-square test, *p* = 0.0114). Therefore, sex was included as a covariate in the multivariable analyses. Body mass index (BMI) was significantly higher in the proteinuria group than in the control group.

The mean BMI was 25.05 ± 4.17 kg/m^2^ in the control group and 28.79 ± 5.20 kg/m^2^ in the proteinuria group. The mean between-group difference was 3.74 kg/m^2^, indicating a significantly higher BMI in the proteinuria group than in the control group (mean difference: 3.74 kg/m^2^; 95% CI: 1.93–5.55; t = 4.09; degrees of freedom = 104; *p* < 0.001). Therefore, BMI was included as a potential confounder in the multivariable models evaluating GBEF and impaired gallbladder emptying. Although a family history of gallstones was more frequently observed in the proteinuria group than in the control group, this difference did not reach statistical significance (17.3% vs. 5.6%, respectively; Fisher’s exact test, *p* = 0.0702).

**Table 1:** Demographic, clinical, laboratory, and ultrasonographic characteristics of the study population.

Variable	Control Group, *n* = 54	Proteinuria Group, *n* = 52	*p* Value	Statistical Test
**Demographic and clinical characteristics**				
Age, years	36.33 ± 11.22	41.33 ± 8.86	0.012	Welch’s t
Female sex, n (%)	38/54 (70.4%)	24/52 (46.2%)	0.011	Pearson’s chi-square
Body mass index, kg/m^2^	25.05 ± 4.17	28.79 ± 5.20	<0.001	Student’s t
Hypertension, n (%)	3/54 (5.6%)	20/52 (38.5%)	<0.001	Pearson’s chi-square
Systolic blood pressure, mmHg	111.11 ± 10.89	118.65 ± 14.11	0.003	Welch’s t
Diastolic blood pressure, mmHg	69.17 ± 7.88	73.94 ± 11.81	0.017	Welch’s t
Family history of gallstones, n (%)	3/54 (5.6%)	9/52 (17.3%)	0.070	Fisher’s exact
**Renal and biochemical characteristics**				
Serum creatinine, mg/dL	0.82 ± 0.17	0.93 ± 0.22	0.010	Welch’s t
eGFR, mL/min/1.73 m^2^	100.76 ± 16.74	90.67 ± 20.93	0.007	Welch’s t
Urine protein/creatinine ratio, mg/g	74.00 [54.00–100.00]	1581.00 [790.50–3775.00]	<0.001	WMW
Serum albumin, g/L	45.14 ± 3.03	35.98 ± 10.29	<0.001	Welch’s t
Serum total protein, g/L	70.61 ± 3.74	63.86 ± 13.82	0.001	Welch’s t
**Ultrasonographic gallbladder parameters**				
Fasting gallbladder volume (V0), mm^3^	24,008 ± 10,929	29,103 ± 17,523	0.077	Welch’s t
Postprandial gallbladder volume at 45 min (V45), mm^3^	9654 [6430–13,179]	13,375 [9469–18,978]	0.0003	WMW
GBEF, %	53.10 ± 20.22%	41.76 ± 19.64%	0.0042	Student’s t
**Proteinuria-group characteristics**				
Primary glomerulonephritis, n (%)	—	45/52 (86.5%)	—	
AA amyloidosis, n (%)	—	7/52 (13.5%)	—	
Non-nephrotic proteinuria, n (%)	—	38/52 (73.1%)	—	
Nephrotic-range proteinuria, n (%)	—	14/52 (26.9%)	—	
Immunosuppressive therapy within the preceding 3 months, n (%)	—	9/52 (17.3%)	—	

Values are presented as mean ± standard deviation, median [IQR, Q1–Q3], or n (%), as appropriate. Abbreviations: AA, amyloid A; eGFR, estimated glomerular filtration rate; GBEF, gallbladder ejection fraction; IQR, interquartile range; SD, standard deviation; V0, fasting gallbladder volume; V45, 45 min postprandial gallbladder volume; WMW, Wilcoxon–Mann–Whitney test.

### 3.2. Gallbladder Volumes and Ejection Fraction in Healthy Controls and Patients with Proteinuria

Fasting gallbladder volume was higher in the proteinuria group than in the control group; however, this difference was not statistically significant. The V0 value was 29,102.83 ± 17,523.21 mm^3^ in the proteinuria group and 24,008.07 ± 10,929.44 mm^3^ in the control group (Welch two-sample *t*-test, *p* = 0.0773) (Table 1).

Postprandial gallbladder volume at 45 min was significantly higher in the proteinuria group than in the control group. The V45 value was 10,346.89 ± 5254.17 mm^3^ in the control group and 15,614.04 ± 9148.35 mm^3^ in the proteinuria group. Because V45 values were not normally distributed and homogeneity of variance was not satisfied, between-group comparison was performed using the Wilcoxon–Mann–Whitney test. The analysis showed that residual gallbladder volume at 45 min was significantly higher in the proteinuria group (*p* = 0.0003).

**Figure 1:**
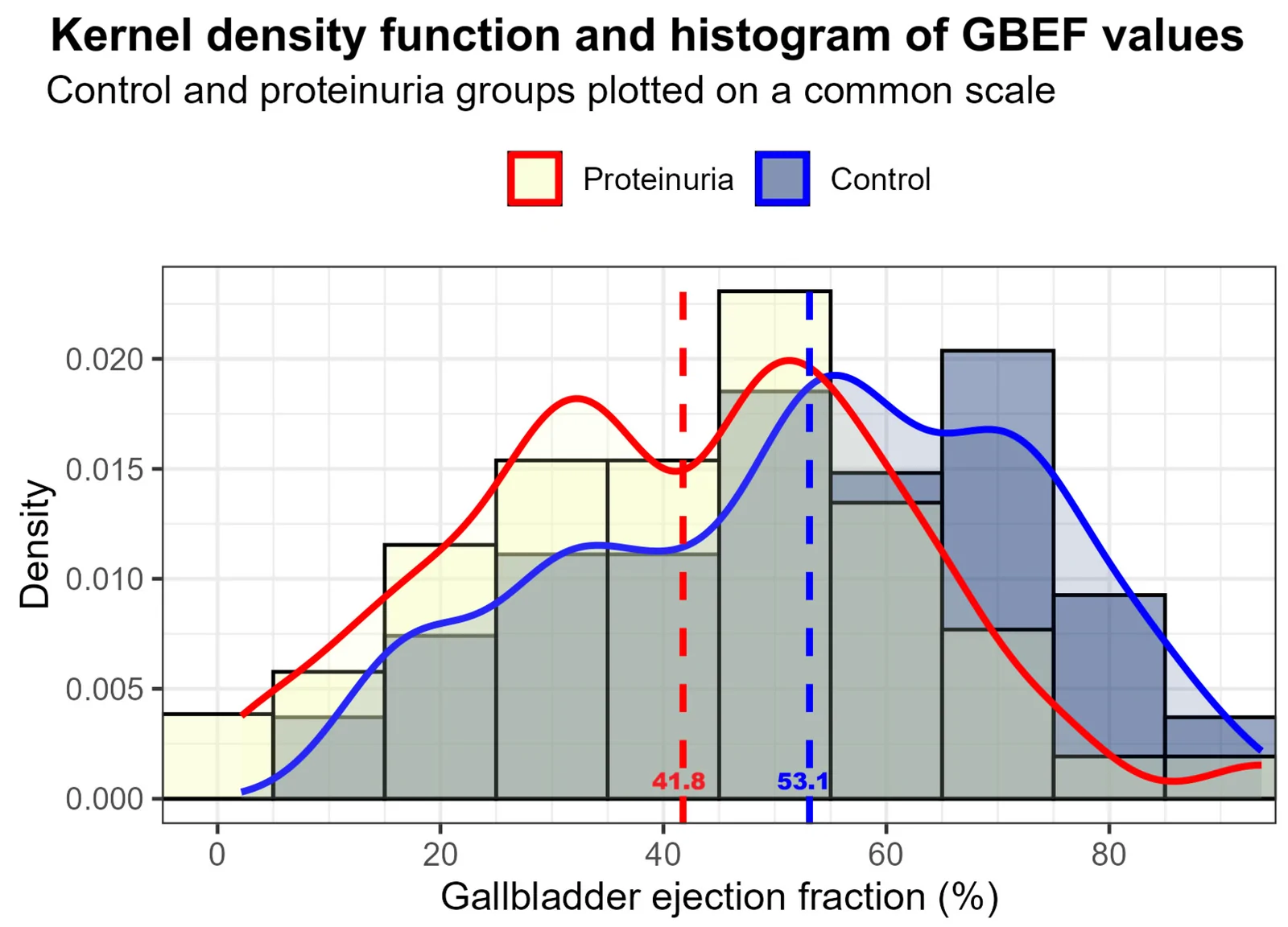
Distribution of GBEF (%) values in healthy controls and patients with proteinuria. [Overlaid histograms and kernel density curves show the distribution of GBEF values in the control and proteinuria groups on a common density scale. Dashed vertical lines indicate the group-specific mean GBEF (%) values].

Gallbladder ejection fraction was significantly lower in the proteinuria group than in healthy controls. The mean GBEF was 53.10 ± 20.22% in the control group and 41.76 ± 19.64% in the proteinuria group (Table 1). Since GBEF values were normally distributed in both groups and homogeneity of variance assumption was met, between-group comparison was performed using the independent-samples Student’s *t*-test. The mean GBEF in the proteinuria group was 11.34 percentage points lower than that in the control group, and this difference was statistically significant (mean difference: −11.34 percentage points; 95% CI: −19.02 to −3.66; t = −2.93; degrees of freedom = 104; *p* = 0.0042) (Figure 1).

In the proteinuria group, 9 of 52 patients (17.3%) had been exposed to immunosuppressive therapy within the 3 months preceding the ultrasonographic gallbladder motility assessment. Among these 9 patients, GBEF <40% was observed in 4 patients (44.4%). The mean V0 value in these 9 patients was 31,333.89 ± 11,955.3 mm^3^, whereas the mean V45 value was 18,063.56 ± 9218.097 mm^3^, and the mean GBEF value was 39.81 ± 23.78%. Because the number of patients receiving immunosuppressive therapy was limited and the number of events was low, motility data were presented descriptively; no statistical comparison was performed between patients who did and did not receive immunosuppressive therapy.

### 3.3. ROC Analysis for Gallbladder Ejection Fraction

**Figure 2:**
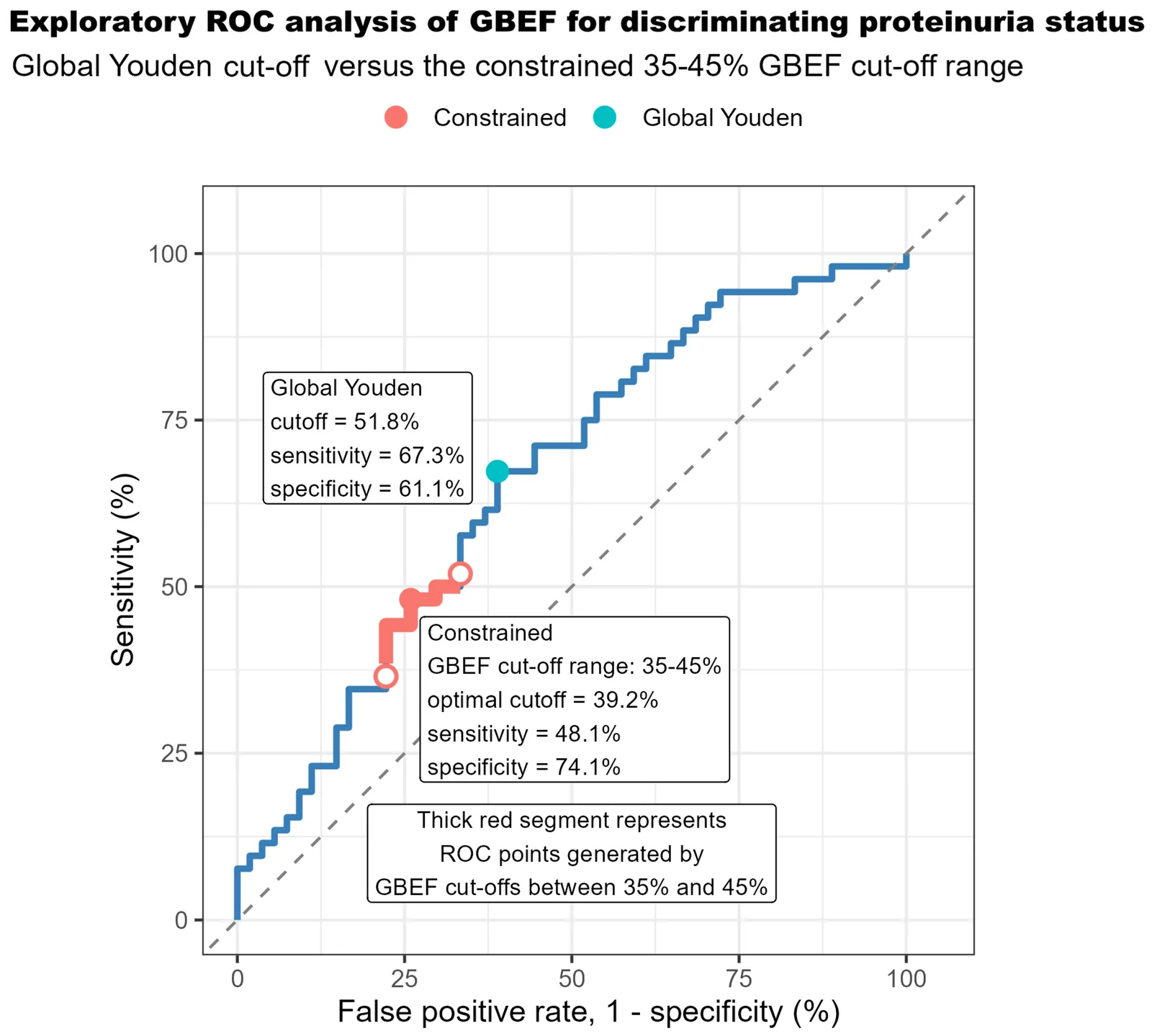
Exploratory ROC analysis of GBEF for discriminating patients with proteinuria from healthy controls. [Proteinuria status was used as the binary reference variable, and GBEF was used as the continuous marker. The unrestricted ROC analysis identified a global Youden cut-off of 51.8% with a sensitivity of 67.3% and specificity of 61.1%. The red segment indicates the clinically relevant 35–45% GBEF threshold range; within this restricted range, the optimal cut-off was 39.2%, with a sensitivity of 48.1% and specificity of 74.1%. ROC-derived thresholds should be interpreted as cohort-specific exploratory discriminatory thresholds, not as definitive pathological cut-off values for gallbladder dysfunction].

In the exploratory ROC analysis, with proteinuria status serving as the binary reference condition and GBEF as the continuous marker, GBEF provided modest discrimination between the proteinuria and control groups, with an area under the curve (AUC) of 0.661. According to the Youden index, the cohort-specific optimal threshold for distinguishing proteinuric patients from healthy controls was 51.8%. At this threshold, sensitivity was 67.3% and specificity was 61.1%. In the restricted ROC analysis, in which the threshold range was limited to 35–45%, the optimal cut-off value was 39.2%. At this threshold, sensitivity was 48.1% and specificity was 74.1% (Figure 2). These values were sample-specific and should not be interpreted as validated diagnostic or clinical cut-offs.

### 3.4. Gallbladder Motility According to Proteinuria Level and Serum Protein Parameters

When patients with proteinuria were compared according to nephrotic and non-nephrotic proteinuria levels, no statistically significant difference was observed between the groups in terms of V0. The V0 value was 29,247.71 ± 18,582.07 mm^3^ in patients with non-nephrotic proteinuria and 28,709.57 ± 14,887.72 mm^3^ in patients with nephrotic-range proteinuria (Mann–Whitney U test, *p* = 0.83).

Similarly, V45 did not differ significantly between the non-nephrotic and nephrotic proteinuria groups. The V45 value was 15,181.45 ± 9175.31 mm^3^ in the non-nephrotic proteinuria group and 16,788.21 ± 9310.87 mm^3^ in the nephrotic proteinuria group (Mann–Whitney U test, *p* = 0.585).

GBEF was numerically lower in patients with nephrotic-range proteinuria than in those with non-nephrotic proteinuria; however, this difference was not statistically significant. The GBEF value was 38.75 ± 17.27% in the nephrotic proteinuria group and 42.87 ± 20.55% in the non-nephrotic proteinuria group (*p* = 0.5078).

In the analysis stratified by serum albumin level, no significant difference in GBEF was observed between patients with albumin levels <32 g/L and those with albumin levels ≥32 g/L. The corresponding GBEF values were 41.81 ± 17.03% and 41.74 ± 20.64%, respectively (*p* = 0.9913). Similarly, no statistically significant difference in GBEF was found between patients with serum total protein levels <57 g/L and those with levels ≥57 g/L. The GBEF values in these groups were 40.41 ± 17.60% and 42.17 ± 20.40%, respectively (*p* = 0.7889).

### 3.5. Gallbladder Motility According to Histopathological Diagnostic Subgroups

When proteinuric patients were evaluated according to kidney biopsy-proven histopathological diagnostic subgroups, no statistically significant difference was observed between the groups in terms of V0 (Kruskal–Wallis test, *p* = 0.1613). V45 also did not differ significantly according to histopathological diagnostic subgroups (Kruskal–Wallis test, *p* = 0.676). Similarly, no statistically significant difference was found between histopathological diagnostic subgroups in terms of GBEF (Kruskal–Wallis test, *p* = 0.464) (Table 2).

### 3.6. Factors Associated with Impaired Gallbladder Emptying

In the primary analysis,

*GBEF* (%) = *β*_0_ + *β*_1_ *group* + *β*_2_ *age* + *β*_3_ *sex* + *β*_4_ *BMI* + *β*_5_ *GBstone.FH* + *ε*,

the GBEF expressed as a percentage was evaluated as a continuous outcome variable, and the association between proteinuria status and GBEF was analyzed using a multivariable linear regression model adjusted for age, sex, BMI, and family history of gallstones. In the multivariable linear regression model adjusted for age, sex, BMI, and family history of gallstones, the presence of proteinuria was independently associated with lower GBEF. The adjusted mean GBEF was 12.52 percentage points lower in the proteinuria group than in the control group (β = −12.52; 95% CI: −21.37 to −3.67; *p* = 0.006) (Table 3). This finding indicates that the association between proteinuria and lower GBEF persisted after adjustment for clinically relevant covariates, including age, sex, BMI, and family history of gallstones. The model was designed to estimate the adjusted association between proteinuria status and GBEF, not to serve as a predictive model. Low R^2^ reflects limited prediction of individual GBEF values, whereas the prespecified primary analysis addressed adjusted group-level association, not individual-level prediction.

Hypertension was more prevalent in the proteinuria group than in the control group [20/52 (38.5%) vs. 3/54 (5.6%), respectively; *p* < 0.001]. In the extended sensitivity model additionally adjusted for HT, proteinuria status remained associated with lower GBEF (adjusted β, −11.28 percentage points; 95% CI, −20.55 to −2.02; *p* = 0.0175). This estimate was similar in magnitude to that obtained in the primary adjusted model (adjusted β, −12.52 percentage points; 95% CI, −21.37 to −3.67; *p* = 0.0060).

In the secondary clinical analysis, when a GBEF value <40% was defined as the pathological gallbladder emptying (i.e., impaired gallbladder emptying), pathological gallbladder emptying was observed in 40 of the 106 participants (37.7%). Pathological gallbladder emptying was more frequently observed in the proteinuria group than in healthy controls. The rate of GBEF <40% was 48.1% in the proteinuria group and 27.8% in the control group (Pearson’s chi-square test, *p* = 0.031; Fisher’s exact test, *p* = 0.045) (Table 4).

In univariable comparisons, serum total protein levels were significantly lower in individuals with GBEF <40% than in those with GBEF ≥40% (median 68.0 g/L versus 70.4 g/L; *p* = 0.018). In contrast, no statistically significant differences were observed between the GBEF <40% and GBEF ≥40% groups in terms of age, sex, BMI, eGFR, serum albumin level, liver enzymes, bilirubin levels, cholesterol parameters, or CRP level (Table 5).

In the unadjusted analysis [*logit*{*P*(*GBEF*(%) < 40%)} = *f*(*group*)], individuals with proteinuria had 2.41-fold higher odds of impaired gallbladder emptying than controls (unadjusted odds ratio: 2.41; 95% CI: 1.07–5.39; *p* = 0.033); the findings for the examined laboratory variables in univariate screening should be considered only as exploratory finding (Table 6).

**Table 5:** Comparison of participants with GBEF <40% and those with GBEF ≥40%.

Variable	GBEF < 40%	GBEF ≥ 40%	Statistical Test	*p*
Age, year	38.5 [29.8–46.0]	38.5 [30.5–47.8]	Mann–Whitney U	0.662
Sex (male)	17/40, 42.5%	27/66, 40.9%	Pearson’s chi-square	0.872
Family history of gallstones	6/40, 15.0%	6/66, 9.1%	Fisher’s exact	0.362
Proteinuria group	25/40, 62.5%	27/66, 40.9%	Pearson’s chi-square	0.031
Hypertension	11/40, 27.5%	12/66, 18.2%	Pearson’s chi-square	0.259
BMI, kg/m^2^	26.98 ± 5.78	26.82 ± 4.59	Student’s t	0.873
eGFR, mL/min/1.73 m^2^	99.50 ± 21.56	93.58 ± 17.93	Student’s t	0.130
Albumin, g/L	41.95 [38.75–46.00]	43.35 [40.00–45.00]	Mann–Whitney U	0.510
Total protein, g/L	68.00 [61.75–71.00]	70.40 [67.40–74.00]	Mann–Whitney U	0.018
Aminotransferase (AST), U/L	18.50 [15.75–25.00]	17.50 [16.00–21.75]	Mann–Whitney U	0.424
Alanine aminotransferase (ALT), U/L	20.00 [14.00–27.50]	17.00 [13.00–24.00]	Mann–Whitney U	0.080
Alkaline phosphatase (ALP), U/L	64.00 [52.75–79.25]	64.00 [53.00–84.00]	Mann–Whitney U	0.982
γ-glutamyltransferase (GGT), U/L	19.50 [13.00–29.25]	20.00 [14.00–27.75]	Mann–Whitney U	0.932
Total bilirubin, mg/dL	0.50 [0.39–0.60]	0.53 [0.41–0.70]	Mann–Whitney U	0.187
Direct bilirubin, mg/dL	0.10 [0.10–0.20]	0.10 [0.07–0.19]	Mann–Whitney U	0.455
Total cholesterol, mg/dL	196.50 [167.75–229.00]	194.50 [165.75–213.50]	Mann–Whitney U	0.436
Low-density lipoprotein (LDL) cholesterol, mg/dL	120.00 [99.75–151.50]	118.00 [102.25–137.25]	Mann–Whitney U	0.529
High-density lipoprotein (HDL) cholesterol, mg/dL	47.50 [40.00–54.25]	48.50 [41.25–55.00]	Mann–Whitney U	0.529
Triglycerides, mg/dL	140.00 [89.50–213.25]	95.50 [66.25–188.75]	Mann–Whitney U	0.059
CRP, mg/L	2.00 [1.00–4.40]	2.00 [1.00–4.85]	Mann–Whitney U	0.827

**Table 6:** Exploratory comparison of participants with GBEF <40% and GBEF ≥40% via univariable logistic model.

Variable	Scale	Unadjusted Odds Ratio	95% Confidence Interval	*p*
Proteinuria group	case vs. control	2.41	1.07–5.39	0.033
Sex	male vs. female	1.07	0.48–2.37	0.872
Hypertension	present vs. absent	1.71	0.67–4.34	0.262
Family history of gallstones	present vs. absent	1.76	0.53–5.90	0.356
Age	per 10-unit increase	0.90	0.61–1.32	0.584
eGFR, mL/min/1.73 m^2^	per 10 mL/min/1.73 m^2^	1.17	0.95–1.45	0.131
Total protein, g/L	per 10 g/L increase	0.74	0.51–1.07	0.110
ALT, U/L	per 10 U/L increase	1.55	1.09–2.22	0.016
LDL, mg/dL	per 10 mg/dL increase	1.06	0.99–1.15	0.106
Triglycerides, mg/dL	per 50 mg/dL increase	1.09	0.93–1.28	0.266

All associations presented in this table were obtained from separate exploratory univariable logistic regression models. The *p* values were not adjusted for multiple comparisons; therefore, statistically significant findings should be regarded as hypothesis-generating and not as evidence of independent associations.

In the exploratory univariable analyses, ALT was associated with GBEF <40%; each 10 U/L increase in ALT was associated with higher unadjusted odds of GBEF <40% (OR, 1.55; 95% CI, 1.09–2.22; *p* = 0.016). Because alkaline phosphatase (ALP), gamma-glutamyl transferase (GGT), and bilirubin are more directly associated with biliary motility disorders, greater emphasis was placed on these parameters. However, none of them showed a statistically significant univariable association with gallbladder motility in the study population. Since the observed association between ALT and GBEF (%) emerged as an unadjusted finding among multiple exploratory comparisons, it was not interpreted as an independent association and was not used for data-driven covariate selection in the multivariable model.

In the multivariable logistic regression model,

*logit*{*P*(*GBEF* (%) < 40%)} = *β*_0_ + *β*_1_ *group* + *β*_2_ *age* + *β*_3_ *sex* + *β*_4_ *BMI*,

adjusted for age, sex, and BMI, the presence of proteinuria remained independently associated with pathological/impaired gallbladder emptying (adjusted odds ratio: 2.95; 95% CI: 1.17–7.47; *p* = 0.022) (Table 7) with higher odds of GBEF <40% compared to that of the estimate from the model additionally containing family history of gallstones (adjusted OR, 2.81; 95% CI, 1.10–7.19; *p* = 0.032), indicating that removal of GBstone.FH did not materially alter the estimated association.

## 4. Discussion

In this case–control study, postprandial gallbladder emptying was shown to be significantly impaired in individuals with proteinuric glomerular disease and preserved kidney function compared with healthy controls. V45 was higher and GBEF was lower in the proteinuria group. The mean GBEF was 11.34 percentage points lower in the proteinuria group than in the control group, the rate of GBEF <40% was 48.1% versus 27.8%, and in the adjusted analysis, the presence of proteinuria increased the odds of pathological gallbladder emptying by approximately 2.8-fold. The persistence of this association after adjustment for age, sex, BMI, and family history of gallstones suggests that proteinuria may be a marker of systemic/neurohormonal effects related to gallbladder motility.

We included patients with biopsy-proven primary glomerular disease or AA amyloidosis who had preserved kidney function. Thus, we sought to minimize the potential effects of chronic kidney disease (CKD) and uremia on gastrointestinal motility. Indeed, previous studies have reported that, in patients with CKD, reduced clearance of gastrointestinal hormones, autonomic nervous system dysfunction, and the uremic milieu may impair gallbladder motility [12,13]. It has also been shown that, in patients with CKD, CCK is cleared more slowly from the plasma, which may lead to hypercholecystokininemia [14]. However, impaired gallbladder emptying has not been demonstrated consistently in this population, and differences in study populations and measurement protocols limit direct comparison with our proteinuric cohort. Therefore, by excluding patients with an eGFR <60 mL/min/1.73 m^2^, we aimed to reduce confounding related to uremia-related gastrointestinal dysmotility.

Postprandial gallbladder emptying is largely regulated by CCK. CCK is a peptide gastrointestinal hormone secreted by enteroendocrine I cells in the intestinal mucosa. Its release increases in response to fat- and protein-rich nutrients, promoting gallbladder contraction through CCK1 receptors and contributing to relaxation of the sphincter of Oddi [1]. Given its physiological role, CCK-related regulation provides a general theoretical context for considering differences in gallbladder emptying. However, circulating CCK concentrations, urinary CCK excretion, and related regulatory peptides were not measured, and no CCK-related mechanism was evaluated in the present study. Therefore, our findings demonstrate an association between proteinuria status and lower GBEF but do not identify the biological pathway underlying this association. Urinary loss of CCK or related regulatory proteins remains an untested hypothesis.

Gallbladder motility can be assessed using methods such as ultrasonography and cholescintigraphy, and fatty meal stimulation is most commonly used in studies evaluating physiological contraction [15,16,17]. Although the operator-dependent nature of ultrasonography is a limitation, its low cost, ease of application, absence of radiation exposure, and reproducibility provide advantages in clinical studies [7]. In this study, the fact that all measurements were performed by the same specialist, using the same device, and 45 min after stimulation with a standardized 40 g chocolate meal reduced methodological variability; moreover, the expectation of maximal contraction at approximately 45 min postprandially supports the physiological appropriateness of the timing.

Although there is no universally accepted single threshold for GBEF, in ultrasonographic assessments performed with fatty meal stimulation, values below 35–40% at 45–60 min are generally interpreted as indicative of reduced gallbladder contractility [10]. In CCK-stimulated cholescintigraphy, an ejection fraction below 40% is also commonly used as a threshold for abnormal gallbladder function [18]. Within the Rome IV framework, the presence of biliary pain, absence of structural pathology, normal liver enzymes, and an ejection fraction <40% on hepatic iminodiacetic acid analogue scan with cholecystokinin administration (HIDA-CCK) scintigraphy have been associated with hypokinetic biliary dyskinesia [19,20]. Therefore, the use of a GBEF <40% threshold is consistent with the literature. In the proteinuric group, both the higher proportion of participants with low GBEF and the larger gallbladder volume remaining 45 min after standardized fatty meal stimulation suggest an inadequate contractile response to standardized fatty meal stimulation and a potential predisposition to bile stasis.

In the pathogenesis of gallstones, lithogenic bile composition, crystal nucleation, and impaired gallbladder motility are key determinants [21,22]. Impairment of the emptying, absorptive, and secretory functions of the gallbladder predisposes to stone formation; the increased development of biliary sludge and gallstones in conditions associated with reduced gallbladder emptying, such as parenteral nutrition, pregnancy, reduced oral intake, or impaired sympathetic innervation, supports this view [23,24]. Previous studies have examined the relationship between kidney disease and gallstones mainly in CKD and dialysis populations, in which an increased prevalence of gallstones has been reported [25,26,27]. However, in these studies, uremia, dietary changes, metabolic disturbances, and comorbidities represent important confounding factors. Data specific to proteinuria are more limited; Sung Keun Park et al. reported that severe proteinuria was associated with an increased risk of incident gallstone formation and that this association persisted after multivariable adjustment [9]. Our study highlights gallbladder dysmotility as a functional intermediate phenotype in the potential link between proteinuria and gallstone risk. Accordingly, our findings support the hypothesis that at least part of the association between proteinuria and gallstone risk may be mediated through impaired gallbladder emptying and increased residual gallbladder volume.

One source of indirect evidence supporting a potential link between proteinuria and gallbladder pathology is the retrospective case–control study by Lindaberry et al., which evaluated the association between gallbladder mucocele formation and proteinuria in dogs [28]. In that study, dogs with gallbladder mucocele had a significantly higher frequency of urine protein concentrations ≥30 mg/dL on dipstick testing than control dogs. Gallbladder mucocele is characterized by abnormal mucus secretion from the gallbladder epithelium and accumulation of mucus within the gallbladder lumen. The mucocele is associated with dysfunctional biliary processes, including bile stasis, biliary obstruction, and gallbladder distension. Although these findings do not provide direct evidence for proteinuric kidney diseases in humans, they may be interpreted as hypothesis-generating indirect data suggesting a biologically plausible link between proteinuria and gallbladder dysfunction or bile stasis.

In subgroup analyses, no significant difference in GBEF was observed according to nephrotic versus non-nephrotic proteinuria, serum albumin/total protein levels, or histopathological diagnostic groups. This finding suggests that impaired gallbladder emptying may not be linearly related solely to the amount of proteinuria or serum albumin level; rather, other factors such as the duration and selectivity of proteinuria, the profile of lost protein–peptide molecules, inflammatory status, lipid metabolism, and autonomic–neurohormonal regulators may play a role. In the univariable analysis, the finding that serum total protein levels were lower in individuals with GBEF <40% suggests that total protein loss or alterations in protein composition may be related to gallbladder motility, independently of serum albumin levels. The absence of significant differences between the pathological and normal GBEF groups in terms of liver enzymes, bilirubin, cholesterol parameters, and CRP levels suggests that the observed motility impairment does not appear to be attributable to overt cholestasis, hepatocellular injury, systemic inflammation, or conventional lipid parameters. In addition, because the sample size was limited for subgroup analyses, smaller effects may not have been detected. In this study, the number of patients exposed to immunosuppressive therapy was low. Therefore, the potential effect of immunosuppressive therapy on gallbladder emptying function could not be reliably evaluated. Accordingly, immunosuppressive therapy was regarded as a potential confounder in interpreting the relationship between proteinuria and GBEF, although it could not be adequately analyzed in our study.

More broadly, concurrent medication use represents an additional source of residual confounding. Detailed exposure data on individual agents, doses, treatment duration, timing, adherence, and indications were not collected prospectively using standardized definitions and could not be reconstructed reliably from routine clinical records. Moreover, several treatments may reflect the manifestations or severity of the underlying glomerular disease, thereby introducing confounding by indication. Accordingly, the observed association should be interpreted cautiously and not as medication-independent or causal.

Hypertension was markedly more prevalent among participants with proteinuria and may have influenced the magnitude of urinary protein excretion or acted as a marker of renal-disease severity. Excluding all hypertensive participants would have removed more than one-third of the proteinuria group and yielded a less representative patient population. In an extended sensitivity model, additional adjustment for HT produced only modest attenuation of the proteinuria-group coefficient, which remained associated with lower GBEF. Nevertheless, this analysis does not eliminate residual confounding related to the duration and severity of HT, achieved blood-pressure control, target-organ effects, or antihypertensive treatment.

The use of unmatched healthy controls and the resulting baseline imbalances represent important limitations. Although multivariable adjustment and the sensitivity analysis additionally adjusted for hypertension yielded consistent findings, residual confounding cannot be excluded. A prospectively matched design or a prespecified propensity-score approach might have improved balance; however, post hoc matching in this modest-sized cohort could have excluded informative participants and reduced precision without addressing unmeasured confounding. Although all participants had preserved kidney function, residual confounding by differences within this range also remains possible. Accordingly, the findings should be interpreted as adjusted associations rather than causal effects.

In the ROC analysis, the discriminative performance of GBEF was modest; the unrestricted analysis yielded an AUC of 0.661 and a cohort-specific threshold of 51.8%, whereas the restricted analysis within the clinically accepted range of 35–45% identified an optimal threshold of 39.2%. This result supports the consistency of the <40% threshold used in this study with both the literature and the data-driven analysis. However, the modest AUC value indicates that GBEF should not be interpreted as an independent screening test for identifying proteinuric patients, but rather as a phenotype reflecting biliary dysfunction that may accompany proteinuria.

The lower GBEF observed in the proteinuria group may have clinical relevance; however, its diagnostic and prognostic implications cannot be established from the present cross-sectional data. Because biliary symptoms and longitudinal biliary outcomes were not systematically assessed, this finding should currently be regarded as hypothesis-generating and warrants prospective investigation.

The main strengths of our study include the biopsy-proven nature of the glomerular diseases in the patient group, the exclusion of patients with eGFR <60 mL/min/1.73 m^2^, the exclusion of major confounding factors such as cholelithiasis, diabetes mellitus, liver disease, oral contraceptive use, and recent rapid weight loss, and the performance of ultrasonographic measurements under standardized conditions by the same specialist. An important methodological aspect of the study is that all proteinuric patients were evaluated within the first 3 months after diagnosis. Thus, the study cohort consisted of newly diagnosed/early-stage cases of proteinuric kidney disease. With this approach, we aimed to provide a more homogeneous clinical evaluation framework for both proteinuric patient–control comparisons and nephrotic–non-nephrotic subgroup analyses by reducing the potential effects of confounders such as cumulative proteinuria exposure related to disease duration, chronic metabolic effects, and long-term treatment variability. The fact that the proteinuria group was older, had a higher BMI, and differed in sex distribution compared with the control group indicates that multivariable adjustment was methodologically necessary; the persistence of the association after adjustment for these variables supports the view that there is an association between proteinuria and lower GBEF. However, because of the case–control design, our findings should be interpreted as associations and cannot establish a causal relationship between proteinuria and impaired gallbladder emptying. Due to the lack of measurement of serum CCK levels, urinary CCK loss, and other gastrointestinal peptide hormones, we could not directly test whether disruption of CCK-related neurohormonal regulation mediated the association between proteinuria and impaired gallbladder emptying. Other limitations include the assessment of gallbladder motility using a single-session ultrasonographic measurement, the absence of scintigraphic confirmation, and the limited sample size for subgroup analyses.

## 5. Conclusions

In conclusion, postprandial gallbladder emptying was significantly impaired in proteinuric patients with glomerular disease and preserved kidney function compared with healthy controls. The presence of proteinuria remained independently associated with impaired gallbladder emptying, defined as GBEF <40%, after adjustment for clinically relevant confounding factors. These findings suggest that gallbladder dysmotility may represent a potentially relevant functional feature of proteinuric kidney disease; however, the diagnostic and prognostic significance of this cross-sectional observation has not yet been established. In particular, the present study cannot determine whether reduced GBEF contributes to subsequent gallstone formation or other biliary outcomes. Prospective longitudinal studies with larger sample sizes, systematic assessment of biliary symptoms and outcomes, direct measurement of CCK and other gastrointestinal peptide hormones, and detailed characterization of the duration and severity of proteinuria are required to clarify the biological basis and clinical relevance of this association.

## Figures and Tables

**Table 2:** GBEF values according to histopathological diagnostic subgroups in patients with proteinuria (Kruskal–Wallis test, *p* = 0.464).

Renal Biopsy Diagnosis	n	Mean ± SD (%)	Median [IQR] (%)
FSGS	24	37.08 ± 18.07	36.99 [24.03–52.54]
MGN	12	41.21 ± 20.39	40.97 [27.69–53.60]
IgA nephropathy	7	53.95 ± 26.81	64.61 [37.13–66.00]
MCD	2	38.57 ± 3.99	38.57 [37.17–39.98]
AA amyloidosis	7	47.47 ± 15.48	50.50 [36.96–53.97]

Abbreviations: FSGS: focal segmental glomerulosclerosis; MGN: membranous glomerulonephritis; IgA nephropathy: immunoglobulin A nephropathy; MCD: minimal change disease; AA amyloidosis: amyloid A amyloidosis.

**Table 3:** Primary multivariable linear regression model for GBEF (%).

Variable	β Coefficient	Standard Error	95% CI	*p* Value
Group, (0: control; 1: case)	−12.52	4.46	−21.37 to −3.67	0.006
Age, years	0.21	0.22	−0.23 to 0.64	0.350
Sex, (0: female; 1: male)	0.14	4.51	−8.80 to 9.08	0.975
Body mass index, kg/m^2^	0.07	0.46	−0.84 to 0.99	0.875
Family history of gallstones	−1.29	6.70	−14.58 to 12.01	0.848

Controls (healthy people with no proteinuria) = 0; cases (patients with proteinuria) = 1. The control group, female sex, and absence of family history of gallstones were used as reference categories. n = 106; residual standard error = 20.20; R^2^ = 0.088; adjusted R^2^ = 0.042.

**Table 4:** Proportion of participants with impaired gallbladder emptying, defined as GBEF <40%, in the control and proteinuria groups.

Group	GBEF ≥ 40%, n	GBEF < 40%, n	Pathologic (GBEF < 40%) Ratio, n (%)
Control	39	15	15/54 (27.8%)
Proteinuria	27	25	25/52 (48.1%)

Pearson’s chi-square test: *p* = 0.031. GBEF: gallbladder ejection fraction.

**Table 7:** Secondary multivariable logistic regression model for impaired gallbladder emptying, defined as GBEF < 40%.

Variable	Adjusted Odds Ratio	95% Confidence Interval	*p*
Proteinuria group	2.95	1.17–7.47	0.022
Age	0.98	0.93–1.03	0.387
Sex (male vs female)	0.91	0.37–2.20	0.829
BMI, kg/m^2^	0.98	0.90–1.08	0.734

The dependent variable was GBEF <40%, coded as 0 = no and 1 = yes. The model included proteinuria group, age, sex, and BMI. Group was coded as 0 = healthy control and 1 = proteinuria, and sex was coded as 0 = female and 1 = male. The healthy control group and female sex were the reference categories.

## Data Availability

The dataset used for the analyses reported in this study has been deposited in Mendeley Data and is publicly available under the title “Ultrasonographic Gallbladder Emptying Data After a Standardized Chocolate Stimulus in Patients with Proteinuria and Healthy Controls.” Version 2, GBdata v2.xlsx. https://doi.org/10.17632/37y668597s.2.

## Supplementary Materials

The following supporting information can be downloaded at https://www.mdpi.com/article/10.3390/jcm15166267/s1, For the reproducibility of the research, the Supplementary Material includes the R code used to generate Figure 1 and Figure 2, together with the formulas, assumptions, code, and numerical results for the effect-size, post hoc power, minimum detectable effect, and required sample-size analyses based on both the unadjusted comparison of GBEF and the primary adjusted multivariable linear regression model.

## Abbreviations

The following abbreviations are used in this manuscript:

AA	Amyloid A
ALP	Alkaline phosphatase
ALT	Alanine aminotransferase
AST	Aminotransferase
AUC	Area Under Curve
BMI	Body mass index
CCK	Cholecystokinin
CI	Confidence interval
CKD	Chronic kidney disease
CKD-EPI	Chronic Kidney Disease Epidemiology Collaboration
CRP	C-reactive protein
eGFR	Estimated glomerular filtration rate
FSGS	Focal segmental glomerulosclerosis
GBEF	Gallbladder ejection fraction
GBstone.FH	Family history of gallbladder stones
GGT	γ-glutamyltransferase
HDL	High-density lipoprotein
HIDA-CCK	Hepatic iminodiacetic acid analogue scan with cholecystokinin administration
HT	Hypertension
IgA	Immunoglobulin A
IQR	Interquartile range
LDL	Low-density lipoprotein
MCD	Minimal Change Disease
MGN	Membranous glomerulonephritis
OR	Odds ratio
ROC	Receiver operating characteristic
SD	Standard deviation
UPCR	Urine protein-to-creatinine ratio
USG	Ultrasonography
