# Supplementary material for: Proteinuria Is Independently Associated with Impaired Gallbladder Emptying in Biopsy-Proven Glomerular Disease with Preserved Kidney Function: A Case–Control Ultrasonographic Study
Source: J Clin Med. 2026 Aug 13;15(16):6267. doi: 10.3390/jcm15166267 (PMC13513181; doi:10.3390/jcm15166267)
Supplement: Supplementary file 1 [file jcm-15-06267-s001.zip › jcm-4440614-supplementary.pdf]

SUPPLEMENTARY MATERIAL

R Code Used in the Study to Generate the Figures and to Perform the Effect Size, Post Hoc Power, and Required Sample Size Analyses

```
# Load the package for reading Excel files
library(readxl) # read_excel
GBdata <- read_excel("G://Simal//2026 04 SafraKesesi//GBdata v2.xlsx") # SafraKesesi=Gallbladder.
# Keep only the data.frame class (remove the classes tbl_df and tbl)
GBdata <- as.data.frame(GBdata)

head(GBdata)

library(lubridate); library(zoo)
names(GBdata)
```

FIGURES

The code for
Figure 1. Distribution of GBEF (%) values in healthy controls and patients with proteinuria.

```
library(dplyr)
library(ggplot2)

# Multivariable Linear Regression Model
m2 <- lm(GBejectionfracPct ~ group + age + sex + BMI + GBstone.FH, data=GBdata)

summary(m2); confint(m2)

# Create separate dataframes for the GBEF (%) values of the control and proteinuria groups.
GBEF_control <- data.frame(GBejectionfracPct=GBdata$GBejectionfracPct[GBdata$group == 0])
GBEF_case <- data.frame(GBejectionfracPct=GBdata$GBejectionfracPct[GBdata$group == 1])

# Combine the control and proteinuria groups into a single dataframe
BirlesikVc <- dplyr::bind_rows(list(Control=GBEF_control, Proteinuria=GBEF_case), .id="StudyGroup")

mean_df <- BirlesikVc %>% group_by(StudyGroup) %>% summarise(mean_GBEF = mean(GBejectionfracPct, na.rm =
TRUE))
mean_df # Control (healthy)=53.1; Proteinuria(Case; patient)=41.8

# Set the group order explicitly to ensure consistent group order, legend order, and color mapping in the plot
BirlesikVc$StudyGroup <- factor(BirlesikVc$StudyGroup, levels=c("Control","Proteinuria"))

# Determine the common x-axis limits for both groups
aralik <- range(BirlesikVc$GBejectionfracPct, na.rm=TRUE); aralik # min(GBejectionfracPct)=2.132273 max=93.656837
# Round the lower limit down to the nearest multiple of 5
endusuk <- floor(aralik[1] / 5) * 5; endusuk      # 0; min
# Round the upper limit up to the nearest multiple of 5
enyuksek <- ceiling(aralik[2] / 5) * 5; enyuksek  # 95; max
```

```
# Histogram + kernel density graph
mygraph <- ggplot(BirlesikVc, aes(x=GBejectionfracPct, fill=StudyGroup, color=StudyGroup)) +
  geom_histogram(aes(y=after_stat(density)), alpha=0.40, binwidth=10, position="identity",  color="black") +
  geom_density(alpha=0.15, linewidth=1, adjust=0.65) +
  geom_vline(
    data=mean_df,
    aes(xintercept=mean_GBEF, color=StudyGroup),
    linetype = "dashed",
    linewidth = 1,
    show.legend = FALSE
  ) +
  scale_fill_manual(values= c(Control="#002366", Proteinuria="#f7fcb9"), breaks=c("Proteinuria", "Control")) +
  scale_color_manual(values=c(Control="blue", Proteinuria="red"), breaks=c("Proteinuria", "Control")) +
  scale_x_continuous(breaks=seq(endusuk, enyuksek, by=20), minor_breaks=seq(endusuk, enyuksek, by=5),
expand=c(0,0)) +
  labs(
    title="Kernel density function and histogram of GBEF values",
    subtitle="Control and proteinuria groups plotted on a common scale",
    x="Gallbladder ejection fraction (%)", y="Density", fill=NULL, color=NULL) +
  theme_bw(base_size=12) +
 theme(panel.grid.minor.x = element_line(), axis.text.x = element_text(size = 10), legend.position = "top",
    legend.justification = "center", plot.title.position = "plot", plot.title = element_text(face = "bold", hjust = 0),
    plot.subtitle = element_text(hjust = 0))

mygraph

#  Save to a .png file with high resolution
ggsave(filename="GBEFp_histogram_kernel_density_control_vs_proteinuria.png", plot=mygraph, dpi=600, width=5.5,
height=4, units="in")
```

The code for
Figure 2. Exploratory ROC analysis of GBEF for discriminating patients with proteinuria from healthy controls.

```
# Load the package for reading Excel files
library(readxl) # read_excel
GBdata <- read_excel("G://Simal//2026 04 SafraKesesi//GBdata v2.xlsx") # SafraKesesi=Gallbladder.
# Keep only the data.frame class (remove the classes tbl_df and tbl)
GBdata <- as.data.frame(GBdata)

head(GBdata)

library(lubridate); library(zoo)
names(GBdata)

library(dplyr)
library(pROC)
library(ggplot2)

# Prepare the ROC data
roc_data <- GBdata %>% select(group, GBejectionfracPct) %>% filter(!is.na(group), !is.na(GBejectionfracPct))

# ROC object
# group coding: 0=Control, 1=Proteinuria
```

```
# Since lower GBEF values are associated with proteinuria, use direction = ">"
roc_obj <- roc(response=roc_data$group, predictor=roc_data$GBejectionfracPct, levels=c(0,1), direction=">",
percent=TRUE, ci=TRUE)

# All ROC coordinates
roc_all <- coords(roc_obj, x="all", ret=c("threshold","specificity","sensitivity"), transpose=FALSE) %>%
  as.data.frame() %>% mutate(false_positive_rate = 100 - specificity)

# Global Youden cutoff (Determine the global optimal cutoff using the Youden index)
global_best <- coords(roc_obj, x="best", best.method="youden", ret=c("threshold", "specificity", "sensitivity"),
transpose=FALSE) %>%
  as.data.frame() %>%
  mutate(type="Global Youden", false_positive_rate = 100 - specificity)

# Restrict the analysis to the GBEF cutoff range of 35% to 45%
ConstrainedWindow <- roc_all %>%  filter(threshold >= 35, threshold <= 45)

# Calculate the Youden index within the restricted cutoff range
ConstrainedCandidates <- ConstrainedWindow %>% mutate(youden_index=sensitivity+specificity-100,
distance_to_40=abs(threshold - 40))

# Identify the cutoff that maximizes the Youden index within the 35%–45% range.
# If multiple cutoffs have the same Youden index, select the one closest to 40%.
ConstrainedBest <- ConstrainedCandidates %>% filter(youden_index == max(youden_index, na.rm=TRUE)) %>%
  arrange(distance_to_40) %>% slice(1) %>% mutate(type="Constrained")

ConstrainedBest

# Display the boundary points corresponding to GBEF cutoffs of 35% and 45%
ConstrainedBounds <- coords(roc_obj, x=c(35,45), input="threshold", ret=c("threshold", "specificity", "sensitivity"),
transpose=FALSE) %>%
  as.data.frame() %>% mutate(false_positive_rate=100 - specificity, bound_label=paste0("GBEF cutoff = ", threshold, "%"))

# Combine the global and constrained optimal cutoff points
points_df <- bind_rows(global_best %>% select(threshold, specificity, sensitivity, false_positive_rate, type),
                       ConstrainedBest %>% select(threshold, specificity, sensitivity, false_positive_rate, type))

# Create the ROC plot
roc_plot <- ggplot() +

  # Plot the complete ROC curve
  geom_path(data=roc_all, aes(x=false_positive_rate, y=sensitivity), linewidth=1.2, color="#377eb8") +

  # Highlight the ROC segment corresponding to GBEF cutoffs between 35% and 45%
  geom_path(data=ConstrainedWindow, aes(x=false_positive_rate, y=sensitivity), linewidth=2.6, color="#F8766D") +

  # Plot the reference (chance) line
  geom_abline(intercept=0, slope=1, linetype="dashed", color="gray50") +

  # Plot the boundary points at GBEF cutoffs of 35% and 45%
  geom_point(data=ConstrainedBounds, aes(x=false_positive_rate, y=sensitivity), shape=21, fill="white", color="#F8766D",
size=3, stroke=1.2) +
```

```
# Plot the global and constrained optimal cutoff points
geom_point(data=points_df, aes(x=false_positive_rate, y=sensitivity, color=type), size=3.5) +

# Add the label for the constrained optimal cutoff
geom_label(data=ConstrainedBest, aes(x=false_positive_rate, y=sensitivity, label=paste0("Constrained\n", "GBEF cut-off range: 35-45%\n", "optimal cutoff = ", round(threshold, 1), "%\n", "sensitivity = ", round(sensitivity, 1), "%\n", "specificity = ", round(specificity, 1), "%")), nudge_x=1.4, nudge_y=-15, hjust=0, size=3.2, linewidth=0.25, fill="white") +

# Add the label for the global Youden cutoff
geom_label(data=global_best, aes(x=false_positive_rate, y=sensitivity, label=paste0("Global Youden\n", "cutoff = ", round(threshold, 1), "%\n", "sensitivity = ", round(sensitivity, 1), "%\n", "specificity = ", round(specificity, 1), "%")), nudge_x=-35, nudge_y=5, hjust=0, size=3.2, linewidth=0.25, fill="white") +

# Add a note describing the highlighted 35%–45% cutoff range
annotate("label", x=50, y=10, label="Thick red segment represents\nROC points generated by\nGBEF cut-offs between 35% and 45%", size=3.2, fill="white", linewidth=0.25) +
scale_color_manual(values=c("Constrained"="#F8766D", "Global Youden"="#00BFC4")) +

labs(title="Exploratory ROC analysis of GBEF for discriminating proteinuria status",
    subtitle="Global Youden cut-off versus the constrained 35-45% GBEF cut-off range",
    x="False positive rate, 1 - specificity (%)",
    y="Sensitivity (%)", color=NULL) +

coord_equal(xlim=c(0, 105), ylim=c(0,105)) +
theme_bw(base_size=12) +
theme(legend.position="top", legend.justification="center", plot.title.position="plot", plot.title=element_text(face="bold", hjust=0), plot.subtitle=element_text(hjust=0), plot.margin=margin(t=8, r=14, b=8, l=8))

#  theme(legend.position="top", plot.title=element_text(face="bold"), plot.title.position="plot")

roc_plot

#  Save to a .png file with high resolution
ggsave(filename="ROC_GBEF_global_vs_the_Constrained_35_45.png", plot=roc_plot, dpi=600, width=9, height=5.8, units="in")
```

EFFECT SIZE, POST HOC POWER, AND REQUIRED SAMPLE SIZE ANALYSES

The following analyses were conducted for **the unadjusted between-group comparison of gallbladder ejection fraction (GBEF) as a continuous outcome**. The calculations were based on a two-sided, equal-variance independent-samples t-test. Accordingly, the post hoc power estimate reported below pertains specifically to **the unadjusted comparison of mean GBEF between the healthy control and proteinuria groups** and does not represent the statistical power of the adjusted multivariable linear regression model.

We will calculate the statistical power of **the adjusted multivariable linear regression model** as well.

(A) The post hoc power estimate of the unadjusted comparison of mean GBEF between the healthy control and proteinuria groups

The observed group-specific standard deviations were highly similar and Levene's test did not indicate heterogeneity of variance between the groups. Therefore, the equal-variance assumption was considered reasonable for this calculation. However, similarity between the observed standard deviations does not establish exact equality of the underlying population variances.

```
# 1. Input values
n1 <- 54 # The number of participants in the first group (healthy control group)
n2 <- 52 # The number of participants in the second group (the proteinuria group)

mean1 <- 53.1034849463 # Mean GBEF (%) in the healthy control group
mean2 <- 41.7604159787 # Mean GBEF (%) in the proteinuria group

sd1 <- 20.2206544476 # Standard deviation of GBEF in the healthy control group
sd2 <- 19.6387920019 # Standard deviation of GBEF in the proteinuria group

alpha <- 0.05 # Two-sided significance level
target_power <- 0.80

# 2. Assessment of the equal-variance assumption
# Ratio of the observed standard deviations: SD ratio = SD1 / SD2
sd_ratio <- sd1 / sd2; sd_ratio # 1.029628

# Ratio of the observed variances: Variance ratio = SD1^2 / SD2^2
variance_ratio <- sd1^2 / sd2^2; variance_ratio # 1.060134
```

The observed standard deviations differed by approximately 3%, and the observed variance ratio was approximately 1.06. These values indicate that the group-specific dispersions were highly similar and support the reasonableness of the equal-variance assumption for this calculation. Nevertheless, similarity of the observed variances does not prove exact equality of the corresponding population variances.

# 3. Pooled standard deviation

Under the equal-variance assumption, the pooled standard deviation is: $SD_{pooled} \equiv \sqrt{\frac{(n_1-1)SD_1^2+(n_2-1)SD_2^2}{n_1+n_2-2}}$

```
pooled_sd <- sqrt(  ((n1 - 1) * sd1^2 + (n2 - 1) * sd2^2) / (n1 + n2 - 2)); pooled_sd # 19.93744
```

```
# 4. Standardized effect size: Cohen's d
# The unadjusted between-group mean difference is: mean GBEF in controls - mean GBEF in patients
mean_difference <- mean1 - mean2; mean_difference # 11.34307
```

# Cohen's d = Mean difference / SD_pooled
d <- mean_difference / pooled_sd; d # 0.5689331
# A positive Cohen's d indicates that mean GBEF was higher in the healthy control group than in the proteinuria group.

# 5. Post hoc power for the observed sample
# Degrees of freedom for the equal-variance independent-samples t-test: df = n1 + n2 - 2
df <- n1 + n2 - 2; df # 104

# Noncentrality parameter for the t-test of two independent groups under the alternative hypothesis H_1; $\delta \equiv d\sqrt{\frac{n_1 n_2}{n_1+n_2}}$
ncp <- d * sqrt((n1 * n2) / (n1 + n2)); ncp # 2.928241

# For a two-sided test with alpha = 0.05, the critical t value is:
t_critical <- qt(1 - alpha / 2, df=df); t_critical # $\alpha = 0.05; \frac{\alpha}{2} = 0.025; t_{0.975,104} = 1.983038$.
# The alternative hypothesis H_1 is hold (the null hypothesis H_0 is rejected)
# when the test statistic falls in either of the following critical regions: $T < -1.98303753$ or $T > 1.98303753$.

Power Calculation (Exact power under the noncentral t distribution).
Post hoc power conditional on the observed effect size under the alternative hypothesis H_1:
The test statistic T follows a noncentral t distribution with df=104 and δ=2.92824087.
The test statistic T under the alternative hypothesis H_1: $T \sim t_{\text{noncentral}}(df = 104, \delta = 2.92824087)$.
Statistical power is the probability that the test statistic falls in either of the two critical regions when the alternative
hypothesis H_1 is hold and the true effect is equal to the observed effect magnitude:
Power = $P(T < -1.98303753) + P(T > 1.98303753)$.

# From the noncentral t distribution: $P(T < -1.98303753) = 0.000000582$; $P(T > 1.98303753) = 0.826681970$.
power_left <- pt(-t_critical, df=df, ncp=ncp); power_left # 5.823357e-07 ≈ 0.000000582.
power_right <- 1 - pt(t_critical, df=df, ncp=ncp); power_right # 0.826681970
power_observed <- power_left + power_right # Power = $0.000000582 + 0.826681970 = 0.826682552$.
power_observed; power_observed * 100 # 0.8266826; 82.66826%

Therefore, the power is 82.66826%. This is a post hoc power estimate conditional on the observed effect size.
It pertains to the unadjusted, equal-variance comparison of continuous GBEF values and
not to the adjusted multivariable linear regression model.

# 6. Required sample size for at least 80% power
The required sample size was evaluated under the following assumptions:
1. Two independent groups; 2. Continuous GBEF outcome; 3. Two-sided hypothesis test; 4. α=0.05; 5. Target power = 0.80;
6. True standardized mean difference equal to the observed Cohen's d; 7. Equal allocation between groups: n1 = n2 = n;
8. Equal population variances; 9. No missing or non-evaluable observations.
Therefore, the resulting sample size represents the required number of evaluable participants per group.

# 6.1. Normal-approximation sample size

# For two equally sized independent groups, an approximate sample size per group is $n_{approx} = \frac{2\left(z_{1-\frac{\alpha}{2}}+z_{1-\beta}\right)^2}{d^2}$.
z_alpha <- qnorm(1 - alpha/2); z_alpha # 1.959964
z_power <- qnorm(target_power); z_power # 0.8416212
n_normal_approximation <- (2 * (z_alpha + z_power)^2) / d^2; n_normal_approximation # 48.49705

The normal approximation yields approximately 48.5 participants per group. Because sample size must be an integer and must not be rounded downward, this approximation would initially suggest $\lceil 48.49705 \rceil = 49$ participants per group.

However, the more accurate noncentral t calculation must be used to determine whether 49 participants actually achieve at least 80% power.

6.2. Noncentral t-based sample size

For equal group sizes, n1 = n2 = n: df = 2n - 2 and $\delta \equiv d\sqrt{\frac{n_1 n_2}{n_1+n_2}} = d\sqrt{\frac{n^2}{2n}} = d\sqrt{\frac{n}{2}}$.

```
# The required sample size is the smallest integer n satisfying: P(T < -t[1-alpha/2, 2n-2]) + P(T >  t[1-alpha/2, 2n-2]) >= 0.80.
power_two_sample_equal_n <- function(n, d, alpha=0.05) {
  df <- 2 * n - 2 # degrees of freedom
  ncp <- d * sqrt(n/2) # noncentrality parameter
  t_critical <- qt(1 - alpha/2, df=df) # the critical t value
  power_left <- pt(-t_critical, df=df, ncp=ncp)
  power_right <- 1 - pt(t_critical, df=df, ncp=ncp)
  power_left + power_right
}
```

```
# Power with 49 evaluable participants per group:
power_n49 <- power_two_sample_equal_n(n=49, d=d, alpha=alpha)
power_n49 # 0.7961190
power_n49 * 100 # 79.61190%
```

Therefore, 49 participants per group would provide approximately 79.6% power and would not meet the prespecified target of at least 80% power.

```
# Power with 50 evaluable participants per group:
power_n50 <- power_two_sample_equal_n(n=50, d=d, alpha=alpha)
power_n50 # 0.8042012
power_n50 * 100 # 80.42012%
```

Therefore, 50 participants per group would provide approximately 80.4% power and would meet the target of at least 80% power.

```
# Determine the smallest integer group size that provides at least 80% power under the specified assumptions:
candidate_n <- 2:200 # 2, 3, …, 200
candidate_power <- sapply(candidate_n, power_two_sample_equal_n, d=d, alpha=alpha)
# 0.06487300, 0.08483141, 0.10497218, 0.12509930, 0.14524690, … , 0.99987304, 0.99987998, 0.99988655, 0.99989276, 0.99989864

required_n_per_group <- min(candidate_n[candidate_power >= target_power]); required_n_per_group # 50
# min(50, 51, 52, 53,…, 197, 198, 199, 200)=50

required_total_sample_size <- 2 * required_n_per_group
required_total_sample_size # 100
```

Thus, under a two-sided, equal-variance independent-samples t-test with α=0.05, equal group allocation, and a true standardized mean difference of Cohen's d=0.5689331, the smallest sample size achieving at least 80% power is: 50 evaluable participants per group or 100 evaluable participants in total [50 is the smallest n that satisfies the power condition: $P\left(T < -t_{1-\frac{\alpha}{2},2n-2}\right) + P\left(T > t_{1-\frac{\alpha}{2},2n-2}\right) \geq 0.80$]. This calculation does not include an allowance for missing data, withdrawals, exclusions, or otherwise non-evaluable participants.

Based on the observed group means and standard deviations, the standardized mean difference in GBEF was Cohen's d = 0.569. With 54 healthy controls and 52 proteinuric patients, the post hoc power to detect an unadjusted difference of this magnitude was approximately 82.66826% using a two-sided, equal-variance independent-samples t-test with an α level of

0.05. Under the additional assumption of equal allocation between groups, the normal approximation yielded an estimated requirement of 48.5 evaluable participants per group. However, a noncentral *t*-based calculation showed that 49 participants per group would provide only 79.6% power, whereas 50 participants per group would provide approximately 80.4% power. Therefore, **50 evaluable participants per group** was the minimum sample size required to achieve at least 80% power under the specified assumptions. These calculations pertain to the unadjusted comparison of GBEF as a continuous outcome and not to the adjusted multivariable linear regression model. **For our study; the number of participants in the healthy control group is 54 ≥ 50; the number of participants in the proteinuria group is 52 ≥ 50**. This retrospective calculation should not be interpreted as an a priori justification of the enrolled sample size.

The power analysis based on the comparison of the two group means
- evaluates the magnitude of the between-group difference in GBEF as a continuous outcome;
- reflects the study's sensitivity for detecting the raw, unadjusted difference between the groups, and
- does not quantify the power of the primary regression coefficient for proteinuria after adjustment for age, sex, BMI, and family history of gallstones.

(B) Regression-Based Post Hoc Power, Sensitivity, and Required Sample Size Analyses for the Adjusted Association Between Proteinuria Status and GBEF

The primary inference in this study was based on a multivariable linear regression model evaluating the adjusted association between proteinuria status and gallbladder ejection fraction (GBEF):

$$GBEF\ (\%) = \beta_0 + \beta_1\, group + \beta_2\, age + \beta_3\, sex + \beta_4\, BMI + \beta_5\, GBstone.FH + \varepsilon.$$

The model included N=106 participants and p=5 explanatory variables: group, representing proteinuria status; age; sex; body mass index (BMI); and family history of gallstones. The residual degrees of freedom were therefore $df_{residual} = v = N - p - 1 = 106 - 5 - 1 = 100$. The adjusted regression coefficient for the proteinuria group was $\beta = -12.52$ percentage points, with a standard error (SE) of 4.46. Thus, after adjustment for age, sex, BMI, and family history of gallstones, mean GBEF was estimated to be 12.52 percentage points lower in participants with proteinuria than in healthy controls. The corresponding t statistic was $t = \frac{\beta}{SE} = \frac{-12.52}{4.46} = -2.807$.

```
# Load the package for reading Excel files
library(readxl) # read_excel
GBdata <- read_excel("G://Simal//2026 04 SafraKesesi//GBdata v2.xlsx") # SafraKesesi=Gallbladder.
# Keep only the data.frame class (remove the classes tbl_df and tbl)
GBdata <- as.data.frame(GBdata)
# Multivariable Linear Regression Model
m2 <- lm(GBejectionfracPct ~ group + age + sex + BMI + GBstone.FH, data=GBdata)
# Model dimensions and analysis specifications
N <- nobs(m2); N # 106
alpha <- 0.05 # Two-sided significance level
p <- length(coef(m2)) - 1; p # 5; the number of explanatory variables in the full model
# One regression coefficient is being tested: the coefficient for group (proteinuria status)
u <- 1 # the number of regression coefficients being tested
# Residual/denominator degrees of freedom: v = N - p – 1 = 106 – 5 – 1 = 100
v <- df.residual(m2) # 100
coefficient_table <- summary(m2)$coefficients
# Coefficient-level results for group (proteinuria status)
beta_group <- coefficient_table["group", "Estimate"] # –12.522263
se_group <- coefficient_table["group", "Std. Error"] # 4.461063
t_group <- coefficient_table["group", "t value"] # –2.807013
p_group <- coefficient_table["group", "Pr(>|t|)"] # 0.006011947
```

Model-specific partial effect size for proteinuria:

The magnitude of the proteinuria-specific effect within the adjusted model is quantified using partial R^2. For a single regression coefficient, partial R^2 can be calculated from its t statistic and the residual degrees of freedom:

$$R^2_{\text{partial}} = \frac{t^2}{t^2 + df_{\text{residual}}}.$$

Substituting the observed values,

$$R^2_{\text{partial,observed}} = \frac{t^2}{t^2 + df_{\text{residual}}} = \frac{(-2.807)^2}{(-2.807)^2 + 100} = \frac{7.880}{107.880} \approx 0.073.$$

# R^2_{partial} of group with the outcome (GBejectionfracPct)
library("sensemakr")
# Observed proteinuria-specific partial effect size
partial_r2(m2, covariates="group") # $R^2_{\text{partial,observed}} = 0.07303832$

$R^2_{\text{partial,observed}} = 0.07304$ indicates that adding group (proteinuria status) to the reduced model accounted for approximately 7.3% of the residual variation in GBEF that remained after age, sex, BMI, and family history of gallstones had been included in the model.

The corresponding Cohen's $f^2_{\text{partial,observed}}$ is

$$f^2_{\text{partial,observed}} = \frac{R^2_{\text{partial,observed}}}{1 - R^2_{\text{partial,observed}}} = \frac{\dfrac{t^2}{t^2 + df_{\text{residual}}}}{1 - \dfrac{t^2}{t^2 + df_{\text{residual}}}} = \frac{t^2}{df_{\text{residual}}}$$
$$= \frac{0.073}{1 - 0.073} \approx 0.07879324 \left\{ = \frac{(-2.807)^2}{100} \right\}.$$

# $f^2_{\text{partial,observed}}$ is a common measure of effect size that can be used directly for sensitivity analysis
partial.f2.observed <- partial_f2(m2, covariates="group") # $f^2_{\text{partial,observed}} = 0.07879324$

The observed proteinuria-specific partial effect was quantified using $R^2_{\text{partial,observed}}$ and Cohen's $f^2_{\text{partial,observed}}$. $R^2_{\text{partial,observed}} = 0.07303832$ indicates that proteinuria status uniquely accounted for approximately 7.3% of the residual variance in GBEF after adjustment for the other covariates. The corresponding $f^2_{\text{partial,observed}} = 0.07879324$ is the observed proteinuria-specific partial effect size calculated from our model using our dataset.

Post hoc power for the observed partial effect. u=1; v=100; $f^2_{\text{partial,observed}} = 0.07879324$; α=0.05
library(pwr) # pwr.f2.test
observed_power_result <- pwr.f2.test(u=u, v=v, f2=partial.f2.observed, sig.level=alpha, power=NULL)
observed_power <- observed_power_result$power; observed_power # 0.8016861
observed_power * 100 # 80.16861% ≈ 80.17%

Using the Cohen's $f^2_{\text{partial,observed}} = 0.07879324$, a general linear model power analysis with 1 tested coefficient, 100 residual degrees of freedom, and an α level of 0.05 yielded an estimated post hoc power of 80.17%.

Observed power ≈ 80.17%; $f^2_{\text{partial,min}} \approx 0.07845467$; $R^2_{\text{partial,min}} \approx 0.07275$. Thus, the Cohen's $f^2_{\text{partial.observed}} = 0.07879324$ should be distinguished from the detectable Cohen's $f^2_{\text{partial,min}} = 0.07845467$ ($R^2_{\text{partial,min}} = 0.07274731$; $N_{min} = 106$ that we show below): $f^2_{\text{partial.observed}}$ is calculated directly from the fitted regression model, whereas $f^2_{\text{partial,min}}$ is determined by solving for the smallest effect that would achieve the prespecified target power under the selected power-analysis framework. $N_{observed} = 106 \geq N_{min} = 106$.

Required total sample size using f^2_{partial}:

u <- 1
alpha <- 0.05 # Significance level; with u=1, the partial F test is equivalent to a two-sided t test of the group coefficient.

```
target_power <- 0.80
# In the full model: residual v (i.e., residual df) = N – p – 1. Therefore, N = v + p + 1.
p <- 5 # The number of explanatory variables in the full model

# Minimum total sample size required to detect an effect equal to the observed partial Cohen's f^2 with 80% power
required_v_result <- pwr.f2.test(u=u, v=NULL, f2=partial.f2.observed, sig.level=alpha, power=target_power)
required_v_continuous <- required_v_result$v; required_v_continuous # 99.57028
required_N_continuous <- required_v_continuous + p + 1; required_N_continuous # 105.5703
required_N <- ceiling(required_N_continuous); required_N # 106

# Verify the smallest integer total sample size
power_N105 <- pwr.f2.test(u=u, v=105 - p - 1, f2=partial.f2.observed, sig.level=alpha, power=NULL)$power
power_N105 # 0.7977435; i.e., N = 105 ⇒ Power ≈ 79.77%
power_N106 <- pwr.f2.test(u=u, v=106 - p - 1, f2=partial.f2.observed, sig.level=alpha, power=NULL)$power
power_N106 # 0.8016861; i.e., N = 106 ⇒ Power ≈ 80.17%
# Thus, the minimum required total sample size of N = 106 is not merely the result of applying the ceiling() function to the
# continuous estimate of 105.5703. Direct integer-based verification shows that N = 105 provides less than 80% power,
# whereas N = 106 provides more than 80% power, confirming that 106 is the smallest total sample size that meets the
# prespecified power requirement. Assuming a true f^2_partial equal to the observed value of 0.07879, 1 tested regression
# coefficient, p=5 explanatory variables, an α level of 0.05, a target power of 80%, and no missing or non-evaluable
# observations, the estimated minimum total sample size was 106 participants. The observed sample of 106 participants
# therefore met this model-specific requirement.
```

The post hoc power estimate should be interpreted cautiously because it was calculated using the partial effect size observed in the same fitted model. Consequently, it does not provide evidence that is independent of the adjusted regression coefficient, its standard error, p value, or 95% confidence interval. A larger observed partial effect automatically produces a higher post hoc power estimate, whereas a smaller observed effect produces a lower estimate. Therefore, post hoc power cannot establish that the study was adequately powered before data collection and should not be used to compensate for an imprecise effect estimate or a wide confidence interval. The adjusted regression coefficient and its 95% confidence interval remain the primary statistical evidence because they directly describe the magnitude and precision of the adjusted association. The **post hoc power estimate** is presented only as a **supplementary**, **model-specific** description of the sensitivity associated with the observed partial effect.

Minimum detectable effect at 80% power:

A complementary sensitivity analysis was performed to determine the smallest proteinuria-specific effect that the final sample could detect with 80% power. The sensemakr functions were used only to calculate the **observed** partial effect sizes; the subsequent power and **minimum-detectable**-effect analyses were performed using pwr::pwr.f2.test(). With N=106 participants, p=5 explanatory variables, df_{residual}=100 residual degrees of freedom, and a two-sided α level of 0.05,

```
# Minimum detectable effect with 80% power:
library(pwr) # pwr.f2.test
minimum_detectable_f2 <- pwr.f2.test(u=1, v=100, f2=NULL, sig.level=0.05, power=0.80)$f2
minimum_detectable_f2 # f^2_partial,min = 0.07845467

minimum_detectable_partial_r2 <- minimum_detectable_f2 / (1 + minimum_detectable_f2)
minimum_detectable_partial_r2 # R^2_partial,min = f^2_partial,min / (1+f^2_partial,min) = 0.07845467 / (1+0.07845467) = 0.07274731
```

For the fixed sample of N=106 participants, 1 tested coefficient, 100 residual degrees of freedom, and an α level of 0.05, the minimum detectable partial Cohen's f^2 at 80% power was 0.07845. The corresponding minimum detectable partial R^2 was 0.07275. Thus, the model had 80% power to detect a proteinuria-specific effect accounting for approximately 7.3% of the residual variance in GBEF.

In the observed model, the partial effect estimates are $f^2_{\text{partial,observed}} \approx 0.07879324$ and $R^2_{\text{partial,observed}} \approx 0.07303832$.

Cohen's $f^2_{\text{partial,observed}} \approx 0.07879324$ was slightly larger than the minimum detectable value of 0.07845467. Similarly, $R^2_{\text{partial,observed}} \approx 0.07303832$ was slightly larger than the corresponding minimum detectable value of 0.07274731. Accordingly, the observed proteinuria-specific effect was marginally above the 80%-power detection threshold under the selected general linear model framework.

Assuming a true partial Cohen's f^2 equal to the observed value of 0.07879, 1 tested regression coefficient, 5 explanatory variables, an α level of 0.05, a target power of 80%, and no missing or non-evaluable observations, the retrospectively estimated minimum total sample size was 106 participants. The observed total sample size of 106 participants therefore met this model-specific threshold. This calculation should **not** be interpreted as an a priori justification of the enrolled sample size.

Overall, the general linear model analysis yielded an estimated post hoc power of 80.17% for the observed proteinuria-specific partial Cohen's f^2 of 0.07879. For the fixed sample of 106 participants, the minimum detectable partial Cohen's f^2 at 80% power was 0.07845, corresponding to a partial R^2 of 0.07275. Conversely, assuming a true partial effect equal to the observed value, the retrospectively estimated minimum total sample size was 106 participants. Thus, the observed sample met the model-specific 80%-power threshold under the stated assumptions. Nevertheless, because these calculations were based on the observed effect size, they should not be interpreted as an a priori justification of the sample size or as evidence independent of the adjusted regression coefficient and its 95% confidence interval. The **adjusted coefficient** and **confidence interval** remain the primary statistical evidence, whereas the post hoc power and sensitivity analyses provide **supplementary information** about the fitted model's statistical sensitivity.
